# Microspatial Heterogeneities and the Absence of Postmortem Contamination in Alzheimer’s Disease Brain Microbiota: An Alzheimer’s Pathobiome Initiative (AlzPI) Study

**DOI:** 10.3390/microorganisms13040807

**Published:** 2025-04-01

**Authors:** Myat N. Thwe, Yves Moné, Bhaswati Sen, Samuel Czerski, Ahmed Azad, Joshua P. Earl, Donald C. Hall, Garth D. Ehrlich

**Affiliations:** 1 School of Biomedical Engineering, Science and Health Systems, Drexel University, Philadelphia, PA 19104, USA; mt3387@drexel.edu; 2 Department of Microbiology and Immunology, Drexel University College of Medicine, Drexel University, Philadelphia, PA 19102, USA; yam25@drexel.edu (Y.M.); sen.bhaswati@gmail.com (B.S.); samuel.czerski@gmail.com (S.C.); aia38@drexel.edu (A.A.); joshearl1@hotmail.com (J.P.E.);; 3 Center for Genomic Sciences, Institute of Molecular Medicine and Infectious Disease, Drexel University, Philadelphia, PA 19102, USA; 4 Genomic Core Facility, Drexel University, Philadelphia, PA 19102, USA; 5 Drexel Medicine Diagnostics, Drexel University College of Medicine, Drexel University, Philadelphia, PA 19102, USA; 6 Center for Advanced Microbial Processing, Institute of Molecular Medicine and Infectious Disease, Drexel University, Philadelphia, PA 19102, USA; 7 Center for Surgical Infections and Biofilms, Institute of Molecular Medicine and Infectious Disease, Drexel University, Philadelphia, PA 19102, USA; 8 Department of Otolaryngology—Head and Neck Surgery, Drexel University College of Medicine, Drexel University, Philadelphia, PA 19102, USA

**Keywords:** Alzheimer’s disease, microbiome, pathobiome, Dirichlet-multinomial modeling, Bayesian statistics, contamination, DNA sequencing, polymerase chain reaction

## Abstract

The discovery of profound differences in the brain microbiota of Alzheimer’s disease (AD) patients and age-matched controls (AMCs) raised questions of postmortem contamination and bacterial transport processes which could be informed by microspatial heterogeneities. We performed semiquantitative species-specific bacterial analyses on multiple micro biopsies from each of the 30 brain specimens (AD and controls). We trimmed ~1 mm of each specimen’s edges for surface contaminants and made multiple sterile biopsy punches of the resultant core of each specimen. To identify species-specific abundances, we used our validated, semiquantitative, full-length 16S rRNA gene pan-domain amplification protocol followed by high-fidelity circular consensus sequencing performed on a Pacific Biosciences Sequel IIe instrument. Statistical analyses showed no significant increase in bacterial abundance on trimmed surfaces compared to core specimens, including *C. acnes*, the most abundant species previously identified in AD. We did find evidence of substantial bacterial species abundance differences among micro-biopsies obtained from within individual tissue blocks supporting our hypothesis of microspatial heterogeneities. The autopsy brain specimens used in our analyses in this study and our previous publication were not contaminated prior to or postharvesting but we suggest that future microbiological analyses of brain specimens include similar types of edge-core comparison analyses. Further, the species-level bacterial abundance heterogeneities among specimens of the same tissue suggest that multiple symbiotic processes may be occurring.

## 1. Introduction

Alzheimer’s disease (AD) is a progressive neurodegenerative disorder that primarily affects cognitive functions, such as memory, thinking, and behavior. It is the most common cause of dementia in older adults, affecting millions of individuals and posing a significant global health burden [1,2,3,4,5,6].

In AD, there is an abnormal accumulation of amyloid beta-protein (Aβ) in the brain. These Aβ proteins can clump together, and form aggregates known as amyloid plaques, which are a hallmark pathological feature of Alzheimer’s disease. Previously it was believed that these Aβ proteins were the incidental byproduct of the amyloid β protein precursor (APP) with a lack of any inherent physiological function [1,2,3]. However, the discovery that Aβ is an antimicrobial peptide with a natural and highly conserved physiological role as part of the innate immune system provided a mechanistic basis for the development of AD whereby repeated stimulation via chronic infection, such as with biofilm bacteria, would result in the overproduction of the Aβ, leading to aggregation and eventually to the pathognomonic plaque formation associated with AD [2,3,4].

Emergent findings have led to the recognition that there is a brain microbiota comprised of a complex community of microorganisms, including bacteria, fungi, and parasites that reside in the brain tissue and cerebrospinal fluid [1,3,5,7,8,9,10], and multiple studies have linked microbes and their biofilms with neurological, neurosurgical conditions [11,12,13,14], all indicating that the brain is not a sterile site. Moreover, alterations in this brain microbiota, leading to an AD pathobiome, likely play an etiological role in AD [1,2,3,4,5,6,7]. Multiple bacterial species, including *Cutibacterium acnes, Acinetobacter, Chlamydia pneumoniae, and Helicobacter pylori*, and multiple fungal species, such as *Candida and Malassezia* species, have been observed in diseased brains [1,5,6,7,8,9,10,11,12,13,14]. Additional studies have found associations between viral CNS infections, particularly herpes viruses, and various spirochaetes, that are likely contributors to the etiology of AD [5,6]. Thus, chronic infections, in general, are likely causative factors for the activation of the inflammatory pathways that can lead to AD [8].

The concept of a normal brain microbiome, which suggests the presence of microorganisms within the healthy brain, is still a topic of active research and debate in the scientific community [7,9,10]. One of the primary concerns in studying the brain microbiome is the possibility of sample contamination during or following autopsies as they are not performed under sterile conditions. Thus, there is a risk of introducing microorganisms from external sources, such as from elsewhere on the cadaver, the skin, and/or nares of the pathologist, and the environment. Contaminating microorganisms can confound research results, particularly due to the relatively sparse microbiome of the brain, making it difficult to distinguish true brain microbiota from contaminants [1,3,4,5,15].

## 2. Materials and Methods

### 2.1. Brain Tissues Sampling

Frozen postmortem human brain samples were obtained from the University of Arkansas for Medical Sciences (UAMS). Full-length 16S rRNA microbiome analyses were performed on 86 samples obtained from 31 brain specimens from AD (n = 14) and age-matched controls (AMC) (n = 17). All AD cases were diagnosed with Braak stages IV-VI with an average postmortem interval of 8 h. The analyzed sample set included samples collected from the entorhinal cortex, the frontal and temporal lobes. Any contamination of autopsy specimens that had accrued during postmortem handling was evaluated by comparing species-specific bacterial abundances from each specimen’s edge to its core. To minimize any risk of contamination of the specimens, they were processed in Class II Type A/B3 biological safety cabinet such that none of the specimens used for DNA extraction had ever been in touch with a non-sterile surface. The edge specimen was prepared by trimming and pooling all peripheral surfaces with a sterile blade. The remaining core was then sampled repeatedly using sterile punch biopsies. Multiple extraction controls without specimen that went through the same handling and processing steps were included for all experiments.

### 2.2. DNA Extraction

DNA was extracted from all specimens using the DNeasy Blood and Tissue Kit (Qiagen, Hilden, Germany) according to the manufacturer’s recommendation with slight modifications. Samples were incubated overnight at 56 °C with 570 μL of ATL tissue lysis buffer and 30 μL of Proteinase K in Lysing Matrix. Specimens were then bead beaten for 10 min at 1500 rpm in a SPEX 1600 MiniG (Cole-Parmer, Vernon Hills, IL, USA) for 10 min at 1500 Hz and centrifuged for 1 min at 13,000 rpm. DNA was eluted with 200 μL AE elution buffer. DNA were quantified using a NanoDrop 2000 spectrophotometer (Thermo Fisher Scientific, Waltham, MA, USA) [16].

### 2.3. Full Length 16S rRNA Gene Amplification

Species-specific microbiome analyses were performed on all specimens using our validated full-length 16S MCSMRT protocol as described with minor modifications [7,16,17,18]. Barcoded 16S rRNA amplicons were generated through a two-step PCR process conducted in 96-well plates. The initial PCR round comprised approximately 10 µL of total DNA (equivalent to approximately 1–2 µg of DNA) as template. We used the 16S rRNA bacterial primers 27F and 1492R, as previously described (each at a concentration of 0.2 µM), and the 1X Q5^®^ Reaction Buffer Pack (New England Biolab, Ipswich, MA, USA) for the first PCR round. The cycling conditions were as follows: an initial denaturation step at 94 °C for 3 min, followed by 25 cycles of denaturation at 94 °C for 30 s, annealing at 54 °C for 30 s, extension at 72 °C for 2 min, and a final extension step at 72 °C for 5 min. The PCR products were subjected to agarose gel electrophoresis to assess both their quality and size. For the second PCR round, a 50 μL reaction volume was prepared, comprising 2.5 μL of a unique primer pair of Barcoded Universal F/R Primers (Pacific Biosciences, Menlo Park, CA, USA, catalog number 100-629-100), 10 μL of 16S rRNA amplicons obtained from each sample, and 2× KAPA HiFi HotStart ReadyMix (Roche, Basel, Switzerland). The cycling conditions were as follows: an initial denaturation step at 94 °C for 3 min, followed by 8 cycles of denaturation at 94 °C for 15 s, annealing at 64 °C for 15 s, extension at 72 °C for 2 min, and a final extension step at 72 °C for 5 min. After the second amplification, the PCR products were subjected to purification using High Prep PCR (Magbio, Gaithersburg, MD, USA) following the manufacturer’s protocol. Subsequently, the cleaned barcoded 16S rRNA amplicons were quantified using the AccuClear Ultra High Sensitivity dsDNA Quantitation Kit (Biotium, Fremont, CA, USA) with a BioTek^TM^ FLx800TM Microplate Fluorescence Reader (BioTek, Winooski, VT, USA).

### 2.4. High Fidelity Circular Consensus Classification (CCS)

SMRTbell sequencing libraries were prepared using the PacBio SMRTbell Express Template Prep Kit 2.0 (Pacific Biosciences, Menlo Park, CA, USA, PartNumber: 100-938-900) and sequenced on a Pacific Biosciences Sequel IIe. The raw subreads were converted into circular consensus (CCS) reads on instrument and demultiplexed using the command-line version of the Pacific Biosciences’ workflow engine pbcromwell (pb_demux_ccs) within the SMRT Link v10.2.0 [7,16,17,18]. Full-length 16S (FL16S) sequences were then clustered into Operational Taxonomic Units (OTUs) and assigned to species taxonomic levels using the Microbiome Classification by the Single Molecule Real-time Sequencing (MCSMRT) pipeline designed by Earl et al. [17].

### 2.5. Analytical Methodologies

#### 2.5.1. Reagent-Related Contamination and Data Filtering

Four blank extraction controls (comprised of all reagents used during sample processing but without sample input) were processed in the same way as the true biological samples to allow identification of any contamination from reagents or during sample processing before sequencing. Contamination assessment was performed using the R packages decontam (v. 1.22.0) [19] and microDecon (v. 1.0.2) [20]. In decontam, contaminating OTUs were detected based on their occurrence in negative controls compared to biological samples (prevalence method), while in microDecon, the negative controls were used to calculate and remove the number of contaminant reads for each OTU in the actual samples (OTUs not present in the negative controls are unaffected by this method). Infrequent OTUs present in less than 10% of the samples and low abundance OTUs (relative abundance <0.005%) were grouped into a composite feature named “OTU_other”.

#### 2.5.2. Parametric and Non-Parametric Tests

Relative abundances were computed for each specimen at the genus level by normalizing the read data. We used both parametric and non-parametric tests to assess the difference between edge and core specimen abundances. The paired t-test is suitable for comparing two conditions when the data are normally distributed, and it helps assess whether any observed difference is significant [21]. The Mann–Whitney U test works by ranking all the data points from both groups combined and then comparing the ranks of the observations in the two groups. It assesses whether one group tends to have higher values than the other, making it suitable for determining whether edge abundances have higher values than core abundances. The Wilcoxon signed-rank test is an alternative to paired t-tests to determine whether the central tendency (median) of the differences between the paired observations is significantly different from zero [21,22]. When we computed statistics to compare the core and edge specimens of the same sample, we did two computations. In one computation, abundance measurements that were equal to zero were included, while in the other, these measurements were excluded. This approach effectively filters out zero-value data points, which can sometimes represent missing or erroneous data. Moreover, if this filtering process led to all the edge or all the core specimens from a single sample being excluded (due to all their measurements being zero), then that entire sample was removed from the analysis.

#### 2.5.3. Differential Abundance Analysis

The OTU differential abundance was tested using a hierarchical Bayesian modeling approach based on a Dirichlet-multinomial model (DMM) [23], which was designed to account for the compositional structure of microbiome data. As a Bayesian analysis, DMM provides posterior probability distribution (PPD) describing the uncertainty of the relative abundance estimate for each OTU. In other words, the mean of PPD represents the point estimate for a given OTU relative abundance and the variation around that mean describes the certainty in that estimate. To quantify the differences in relative abundance between two sample groups (edge samples compared to core samples or AD samples compared to AMC), the PPD for the OTU of interest in one sample group was subtracted from the PPD of that OTU in the second sample group. Following convention, if 95% of the PPD for difference does not overlap zero, there is high certainty that the OTU of interest differs in relative abundance between the sample groups. DMM was specified in the Stan probabilistic programming language through the Python interface Pystan (version 2.19.1.1) that implements the Hamiltonian Monte Carlo No-U-Turn sampler (HMC-NUTS) algorithm. For each of the four chains, 3500 iterations were used with 1500 burn-in and a total of 4000 samples were drawn (thin = 2). Convergence was assessed using the Gelman-Rubin statistic [24]. Moreover, this Bayesian analysis detects many more true positives, and this increased sensitivity facilitated the detection of subtle shifts in relative abundance between sampling groups with an acceptably low number of false positives [19,23,25]. We chose this method of analysis to identify differences in the species compositional patterns between multiple pairs of specimen types within the dataset, including the following: (1) Comparison of Edge Samples in Alzheimer’s Disease vs. Age-Matched Control Samples; (2) Examination of Core Samples in Alzheimer’s Disease vs. Age-Matched Control Samples; (3) Comprehensive Analysis of All Samples between Alzheimer’s Disease and Age-Matched Control Samples; (4) Discerning Differences between Edge and Core in AD and AMC cohorts.

## 3. Results and Discussion

**Figure 1:**
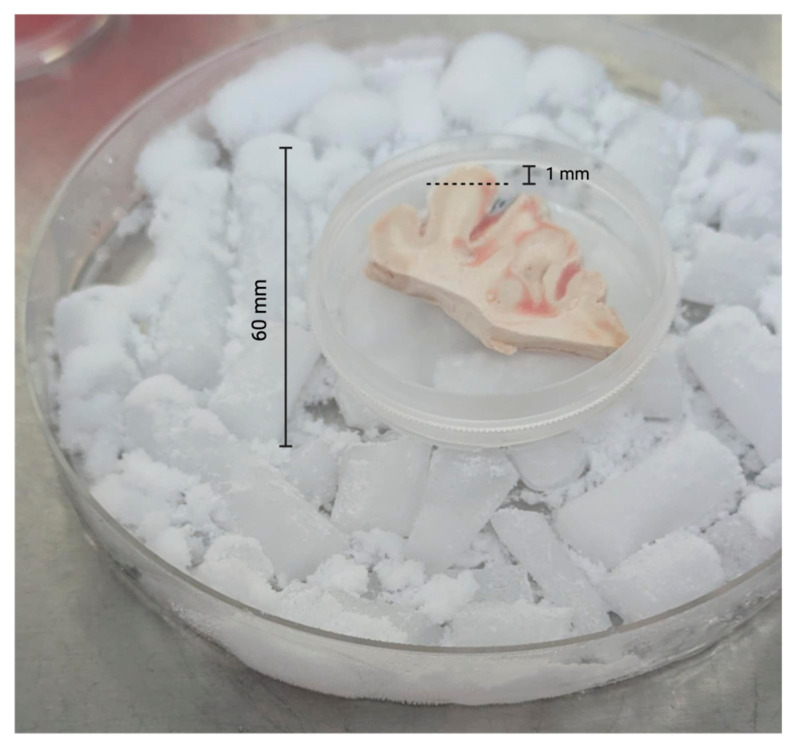
Trimming of brain tissue specimens. The dotted line indicates where the postmortem tissue was trimmed (~1 mm from edge) representing the peripheral surface of the brain tissue sample.

**Figure 2:**
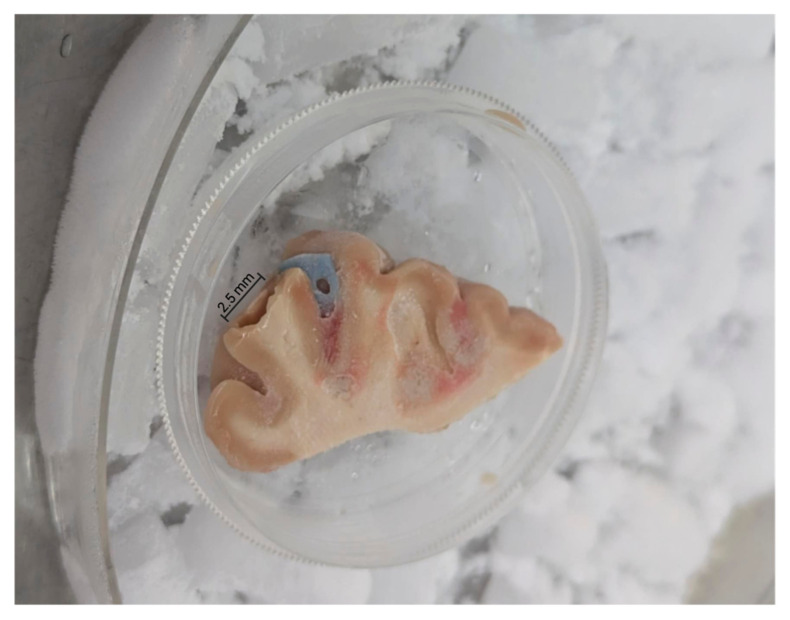
Brain tissue remaining after biopsy punches (2.5 mm) and surface trimming.

To prepare the edge specimens, we trimmed approximately 1 mm from all outer edges of each specimen with sterile razor blades (Figure 1). All edges for all specimens were trimmed in a like manner. After trimming the edges, we used 2.5 mm sterile biopsy punches to obtain multiple central cores of each brain tissue sample (Figure 2).

We performed semiquantitative species-specific bacterial analyses using our validated pan-domain assay [7,16,17,18], which utilizes a full length 16S gene PCR and high-fidelity, single molecule, error-correcting circular consensus sequencing to comparatively analyze the edge and core brain specimens from both AD patients and AMC. The specimens displayed significant heterogeneity with substantial variances in abundance values [17]. Based on prevalence, with abundance > 5% in at least one specimen, the top 30 species are displayed (Table 1). These data are generally consistent with earlier findings concerning the differences in bacterial compositions of the AD pathobiome and the AMC microbiome [7,15]. Three OTUs (OTU_1: *Crinalium epipsammum*, OTU_12: *Pseudomonas thermotolerans*, OUT_272: *Anabaena cylindrica*) were identified as contaminants using decontam [19] and microDecon [20] methods and removed from the entire dataset.

### 3.1. Difference in Relative Abundance Between Edge and Core Brain Specimens

**Figure 3:**
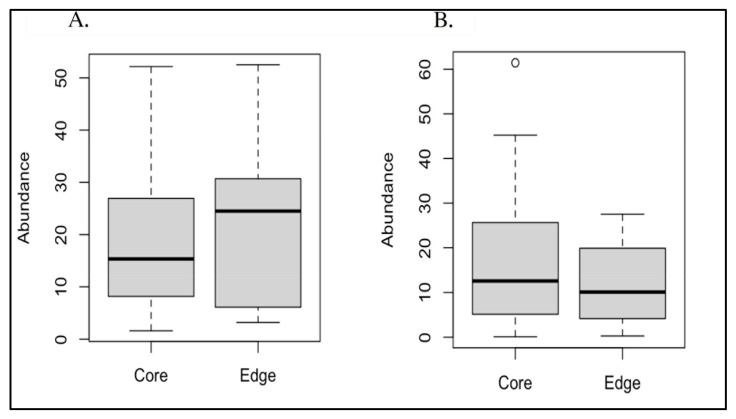
(**A**) Boxplot of core vs. edge abundances for *Acinetobacter*; (**B**) boxplot of core vs. edge abundances for *Cutibacterium acnes*.

Due to their significantly different overall prevalences between AD and AMC brain specimens [7], we conducted paired t-tests to compare both *Acinetobacter* sp. and *C. acnes* abundances between edge and core specimens, resulting in non-significant *p*-values of 0.647 and 0.217, respectively (Figure 3). In addition, we evaluated the edge vs. core data with the Mann–Whitney test and the Wilcoxon signed-rank test, which also both yielded non-significant *p*-values (Table 2).

The results for *Cutibacterium acnes* were consistent between the unfiltered and filtered datasets. Conversely, the results for *Acinetobacter* differed significantly between the unfiltered and filtered datasets. In the unfiltered cases, the results were either borderline or below the statistical significance threshold of *p* = 0.05. However, in the filtered cases, the results for these bacteria were well above this cutoff, indicating a lack of statistical significance.

**Figure 4:**
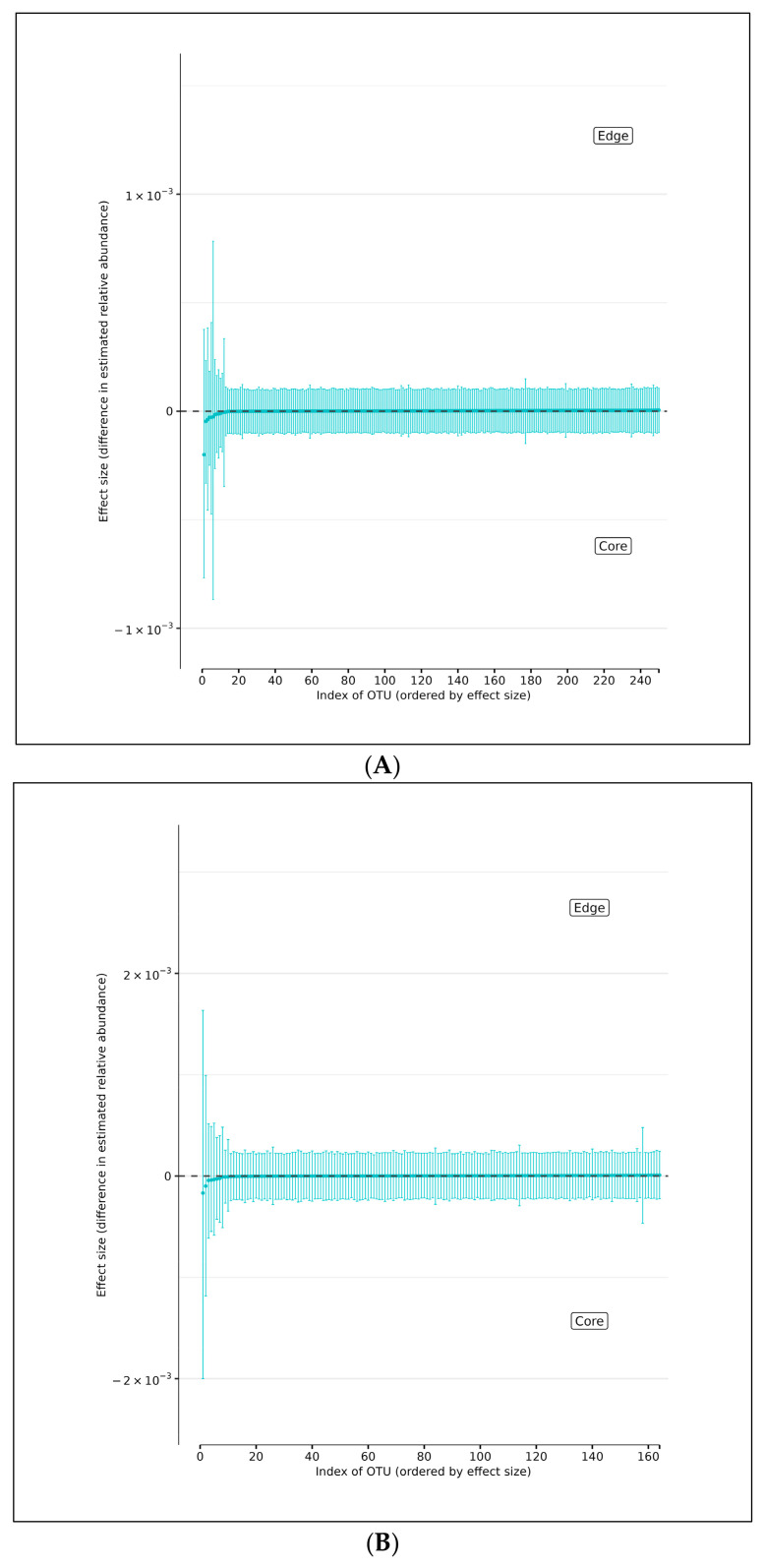

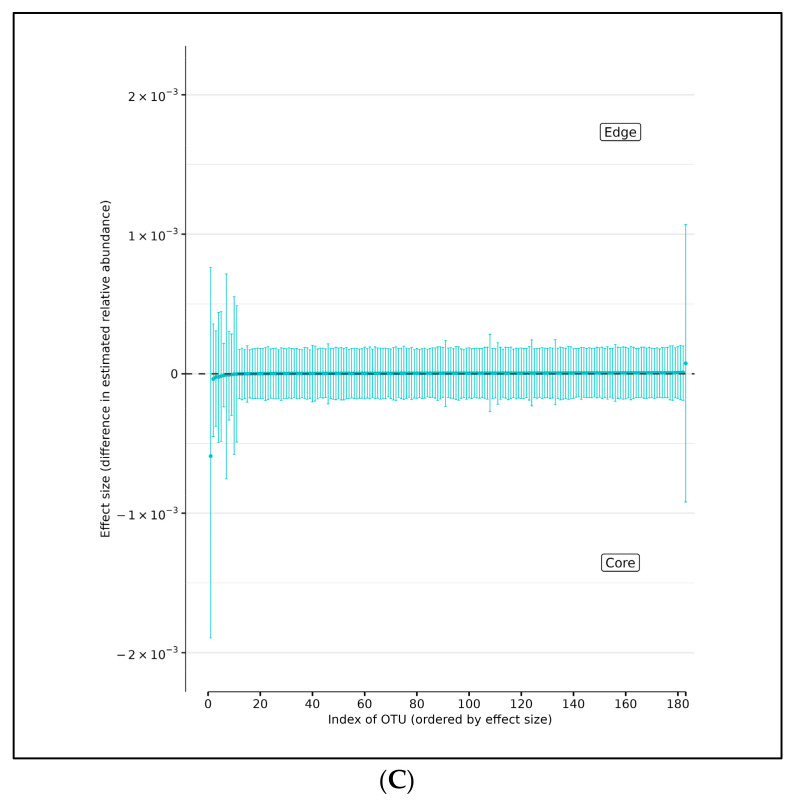
(**A**) Differences in relative abundance between edge and core samples from all brain specimens from both AD and AMC subjects. (**B**) Differences in relative abundance between edge and core samples in brain specimens from individuals with AD. (**C**) Differences in relative abundance between edge and core samples in brain specimens from control (AMC) group. Blue lines indicate no statistically significant difference between the groups.

Using DMM analyses of all edge vs. core abundances from all AD and AMC specimens, we found no evidence of increased bacterial abundance from any OTU level on the surface specimens compared with the core specimens (Figure 4A). Less than 95% of the distribution of differences lies on either side of zero, indicating that there is high certainty that the OTU of interest does not differ in relative abundance between edge and core samples. Similarly, analyses performed individually on edge and core specimens from the AD and AMC cohorts, respectively, showed no appreciable differences in relative abundances either (Figure 4B,C). The relative abundances were estimated for each OTU from each group using DMM. The vertical axis shows the estimated differences in the relative abundance of each OTU between the edge and core groups. Points are the means of the posterior probability distribution of differences (PPD) and the whiskers show the 95% equal-tail probability intervals of PPD (Figure 4A–C).

### 3.2. Differences in Microbial Relative Abundances Between AD and AMC Cohorts

To examine variations in the relative abundances of individual OTUs between edge and core sampling groups, we subjected the OTU count data to analysis employing a hierarchical Bayesian model predicated on the Dirichlet and multinomial distributions. These distributions were employed to construct a hierarchical model for estimating the relative abundances within samples and sampling groups, facilitating the identification of discrepancies in relative abundances for each OTU between edge and core samples in AD and control groups. The results indicate that there were no significant differences in relative abundance between edge and core, which suggests that the specimens used in this analysis and our previous work [7] were not contaminated and that the differences observed between the microbiomes of the AMC subjects and the AD patients are reflective of the development of a pathobiome.

**Figure 5:**
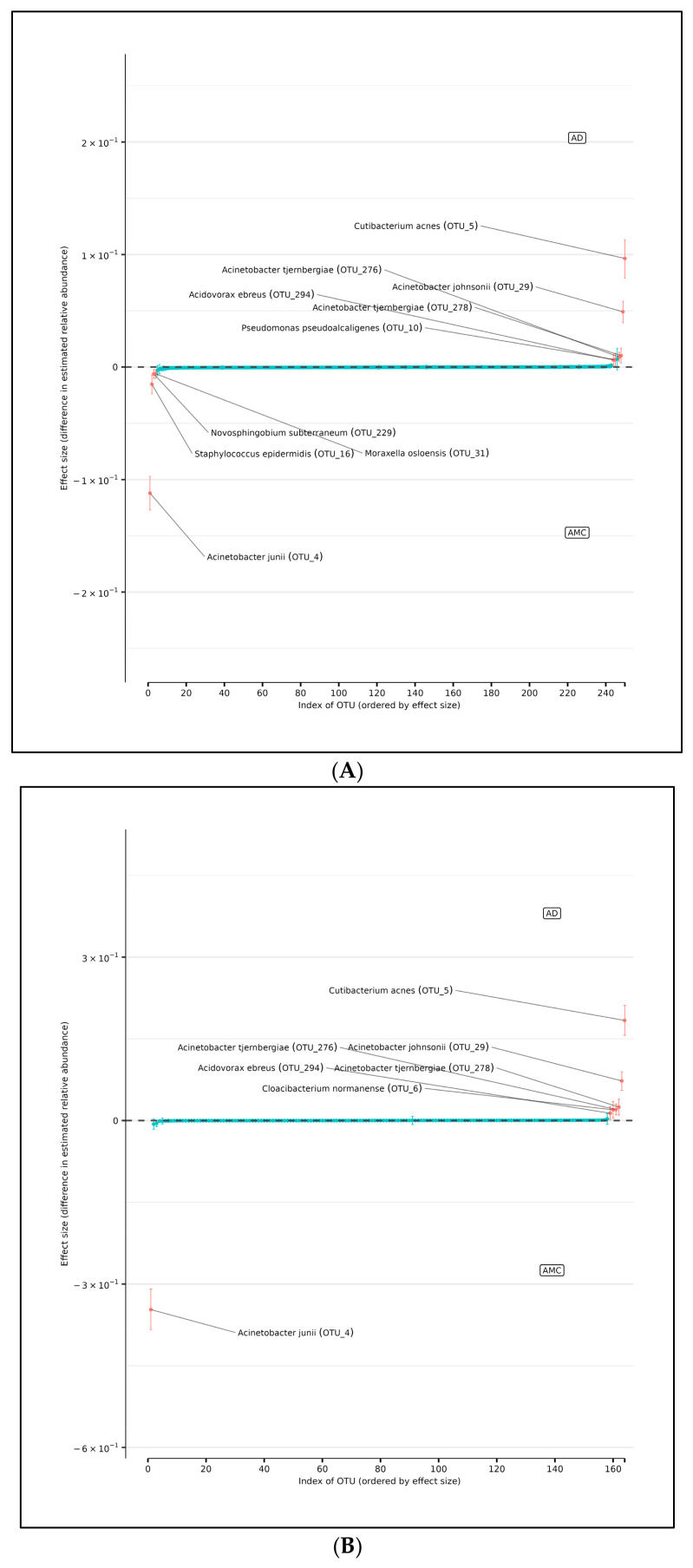

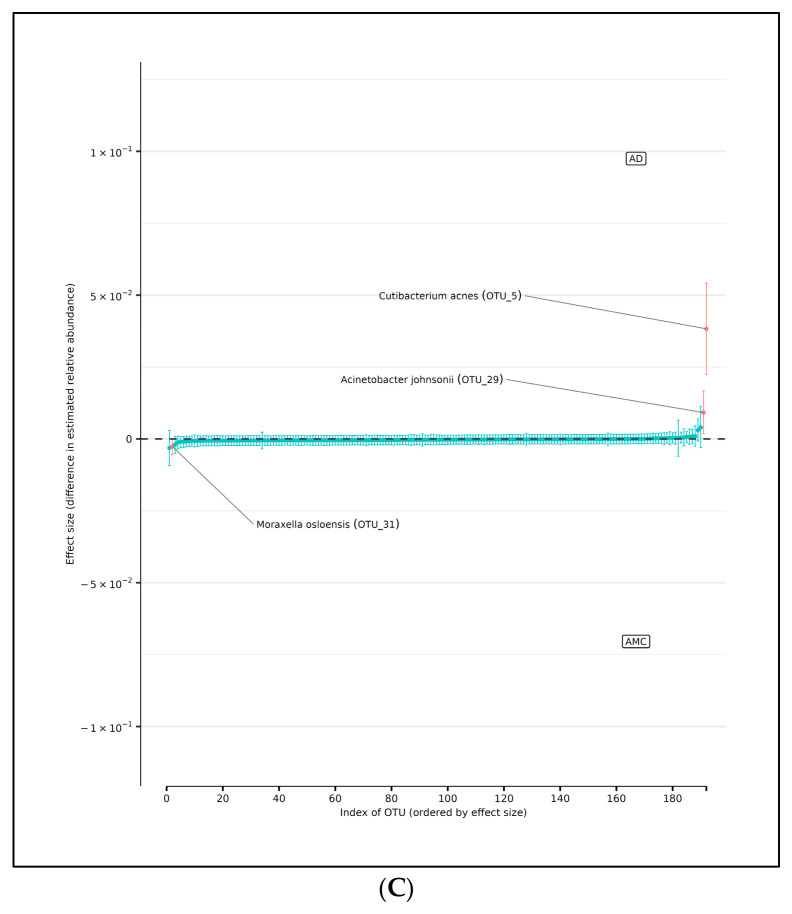
(**A**) Differences in relative abundance of all brain samples between the AD and AMC cohorts. The relative abundances were estimated for each OTU from each group using DMM. The vertical axis shows the estimated differences in the relative abundance of each OTU between the AD and AMC groups. Points are the means of the posterior probability distribution of differences (PPD) and the whiskers show the 95% equal tail probability intervals of PPD. Red dots and lines represent OTU shifted in relative abundance unlike the blue ones. (**B**) Differences in relative abundance on the peripheral surface of the brain samples ‘edge’ between the AD and AMC cohorts. Points are the means of the posterior probability distribution of differences (PPD) and the whiskers show the 95% equal tail probability intervals of PPD. Red dots and lines represent OTU shifted in relative abundance unlike the blue. (**C**) Differences in relative abundance on the biopsy punches of the brain samples ‘core’ between the AD cohort and AMC cohorts. Points are the means of the posterior probability distribution of differences (PPD) and the whiskers show the 95% equal tail probability intervals of PPD. Red dots and lines represent OTU shifted in relative abundance unlike the blue ones. Blue lines indicate no statistically significant difference between the groups. Red dots represent statistically significant differences between the groups.

In support of this conjecture, using DMM and assuming sample non-independence due to multiple samples coming from a single subject in the model, we found 10 OTUs that shift in relative abundance between the AD and AMC cohorts (Figure 5A). Four of these were more abundant in the AMC cohort (*Acinetobacter junii, Staphylococcus epidermidis, Novosphingobium subterraneum,* and *Moraxella osloensis*) and six that were more abundant in the AD cohort: *Acinetobacter tjernbergiae* (OTU_276), *Acinetobacter tjernbergiae* (OTU_278), *Acinetobacter johnsonii, Cutibacterium acnes, and Pseudomonas pseudoalcaligenes*. Note that two different OTU centroids matched *A. tjernbergie* most closely: this likely indicates that one of them is a closely related previously unidentified species.

### 3.3. Differences in Relative Abundance of Edge Samples Between AD and AMC

There are seven OTUs that show abundance differences between AD and AMC edge samples from the DMM analyses, which assume that the samples are non-independent due to multiple samples coming from a single subject. There are six OTUs that were found to be more abundant in the AD cohort than among the AMC: the two *Acinetobacter tjernbergiae*, *Acidovorax ebreus*, *Cloacibacterium normanense*, *Acinetobacter johnsonii*, and *Cutibacterium acnes*. However, there was only one OTU that was more prevalent among the AMC: *Acinetobacter junii* (Figure 5B).

### 3.4. Difference in Relative Abundance of Core Samples Between AD and Controls

Similarly, the DMM analyses showed that there were three OTUs that showed differences in abundance between AD and AMC core brain tissue samples (Figure 5C). Again, we assumed the non-independence of samples due to multiple samples coming from a single specimen and multiple specimens from a subject. There were two OTUs found to be over-represented in the AD group compared to the AMC cohort: *Acinetobacter johnsonii and Cutibacterium acnes*. Similarly, there was one OTU that was more prevalent in the AMC cohort*: Moraxella osloensis.*

## 4. Conclusions

To assess the potential microbial contamination of autopsied brain specimens, we analyzed differences in abundances for the edge (environmentally exposed) and core (non-exposed) of fresh frozen brain specimens from both AD patients and AMCs. We applied both parametric and non-parametric tests for the two most prevalent taxa, *Cutibacterium acnes* and *Acinetobacter* sp., all of which produced non-significant results. Thus, using a battery of statistical tests, there is no indication that specimens used in this analysis and our previous work [7] were contaminated postmortem via harvesting or during handling, shipping, and storage. If there had been any significant contamination during sampling, the peripheral surface of the brain tissue would be expected to have higher microbial abundances of some microbes compared to the core biopsies from the same tissue sample. Moreover, the DMM method reveals that *Cutibacterium acnes, Acinetobacter johnsonii* are associated with AD and prevalent in both edge and core sampling groups. *Acinetobacter junii* was also consistently found in the AMC group in both the edge and across all the specimens. These data are consistent with our previous finding of an increased abundance of *Cutibacterium acnes* being associated with AD and with *Acinetobacter junii* being predominant among members of the AMC cohort [7,26,27].

Future research will include a metaomic investigation of the major AD bacterial pathogens to provide a more complete picture of their interactions and their pathogenic impact on the AD brain. These studies will augment our current analyses examining the microbial burden imposed by the prevalent species (*Cutibacterium acnes and Acinetobacter* sp.,). The use of species-specific DNA and RNA FISH and strain-specific immunohistochemistry with high resolution microscopic techniques to spatially characterize both multispecies aggregates and virulence factor expression will provide detailed mechanistic insights into the microbial contribution to AD pathogenesis.

## Figures and Tables

**Table 1:** Top thirty bacterial genera by overall prevalence among all specimens.

*Acinetobacter*	*Bacillus*
*Cutibacterium*	*Anaerococcus*
*Anabaena*	*Sneathia*
*Cloacibacterium*	*Prevotella*
*Acidovorax*	*Micrococcus*
*Staphylococcus*	*Granulicatella*
*Pseudomonas*	*Virgibacillus*
*Novosphingobium*	*Paracoccus*
*Corynebacterium*	*Pantoea*
*Comamonas*	*Methylobacterium*
*Moraxella*	*Chryseobacterium*
*Streptococcus*	*Bradyrhizobium*
*Klebsiella*	*Bergeyella*
*Sphingomonas*	*Bacteroides*
*Blastomonas*	*Porphyromonas*

Rank: descending prevalence from top to bottom, then left to right.

**Table 2:** Edge vs. core statistics for *Acinetobater* sp. and *Cutibacterium* *acnes* using filtered data.

	One Tailed *t* Test (*p*-Value)	Mann-Whitney (*p*-Value)	Wilcoxon Signed Rank (*p*-Value)
*Acinetobacter* sp.	0.647	0.597	0.745
*Cutibacterium acnes*	0.217	0.509	0.587

## Data Availability

The full-length 16S sequences have been deposited at the NIH NCBI SRA repository (BioProject PRJNA1242302). The datasets generated in this study have been deposited on Github: https://github.com/y-mone/brain-microbiome (accessed on 28 March 2025).
